# Occult Hepatitis B Virus Infection in Maintenance Hemodialysis Patients: Prevalence and Mutations in “a” Determinant

**DOI:** 10.7150/ijms.49540

**Published:** 2020-08-25

**Authors:** Yun Tang, Xiangqin Liu, Xiangheng Lu, Qiang He, Guisen Li, Yang Zou

**Affiliations:** 1 Department of Nephrology, Sichuan Academy of Medical Science and Sichuan Provincial People's Hospital, School of Medicine, University of Electronic Science and Technology of China, Chengdu 610072, Sichuan, China.; 2 Department of Clinical Laboratory, Sichuan Academy of Medical Science and Sichuan Provincial People's Hospital, School of Medicine, University of Electronic Science and Technology of China, Chengdu 610072, Sichuan, China.; 3 School of Medicine, Nanchang University, Nanchang 330047, China.

**Keywords:** Occult hepatitis B virus infection(OBI), maintenance hemodialysis, prevalence, amino acid mutation, genotype

## Abstract

**Background:** Occult hepatitis B virus infection (OBI) is defined as undetectable serum hepatitis B surface antigen (HBsAg) with detectable HBV-DNA in the serum or liver. Patients with maintenance hemodialysis (MHD) are at a high risk of OBI. The prevalence of OBI in MHD patients in China is not well evaluated. In this study, we aim to assess the prevalence of OBI in MHD patients in Sichuan Province, Southwest of China and investigate the mutations in the “a” determinant of HBsAg.

**Methods:** A total of 330 patients undergoing MHD at Sichuan Provincial People's Hospital were enrolled. Serum samples were collected for ELISA assay to test the serological markers of HBV infection, real-time PCR assay to identify the presence of HBV-DNA, and nested PCR plus sequencing analysis to investigate the gene mutations.

**Results:** In a total of 330 MHD patients, we found that the prevalence of OBI was 4.2% (7/165) in the test group, 2.1% (7/330) in the overall dialysis cohort. After a follow-up study of 7 MHD patients with OBI for 2 years, 2 (isolated HBcAb+) of them were still detectable for HBV-DNA. By sequencing analysis, we revealed mutations at the “a” determinant of HBsAg, including Q129R, T131N, M133S, F134L and D144E. The Q129R and M133S mutations were first reported.

**Conclusions:** Our study clarifies the prevalence of OBI in MHD patients in Sichuan Province(4.2% in the test group, 2.1% in the overall dialysis cohort), and demonstrate the mutations of Q129R and M133S in the “a” determinant of HBsAg for the first time.

## Introduction

Chronic hepatitis B virus (HBV) infection is a common cause of liver disease globally, with a high burden. Currently, about 3.5% of the global population is chronically infected with HBV 1, and the infection rate is about 7.18% in China 2. In maintenance hemodialysis (MHD) patients, the infection rate of HBV ranges from 11% to 15% in developing countries 3-5. HBV infections were with multiple clinical presentations, other than chronic HBV infection, occult hepatitis B virus infection (OBI) is also very common. The definition was posted by the Working group of European Association for the Study of the Liver (EASL) in 2008 6. The definition of OBI is the presence of HBV-DNA in the liver or serum with undetectable hepatitis B surface antigen (HBsAg) with or without HBV antibodies, meanwhile serum HBV-DNA level should be less than 200IU/mL, OBI is most frequently seen in patients with isolated hepatitis B core antibody(HBcAb)(+) 7. Reports showed that HBV has been classified into ten genotypes (labeled A-J), different genotypes are associated with different clinical reports, genotype B is more likely to disseminate in hemodialysis environment 8. Patients with MHD are at an increased risk of OBI due to shared dialysis equipment, frequent necessity of blood product transfer invasive procedures, impaired host immune response and lower response to HBV vaccination 7. Globally, the prevalence of OBI among hemodialysis patients ranges from 0% to 58% 9, the explanations of the variation could be differences in methodology, target HBV genomes, and analyzed samples, which probably caused the disparity. However, the prevalence of OBI in MHD patients in China is not well evaluated.

As the exact mechanism of OBI is not clear, several explanations have been proposed. The mutations in the “a” determinant of HBsAg were one of the well-known mechanisms of OBI 10. The HBsAg is encoded by the *S* gene which includes the N-terminal (amino acid positions 1-99), the major hydrophilic region (MHR) (amino acid positions 100-169) and the C-terminal (amino acid positions 170-226). The dominant neutralizing epitope termed the “a” determinant in the major hydrophilic region (MHR) is the key area to enable the antibody to response against HBV infection of HBsAg. It is usually considered to be located between amino acids 124 and 147 or 149 of HBsAg, and mutations in this region are associated with the generation of vaccine escape variants and persistent infection, eventually the HBsAg couldn't be identified by commercially HBsAg test kits, then OBI occurred 11, 12.

In this study, we assessed the prevalence of OBI in MHD patients in Sichuan Province of China. We revealed genomic mutations of in “a” determinant of the S gene. Our work supplements current knowledge of OBI prevalence and its association with MHD in China.

## Methods

### Study design

The study included 330 adult patients who received three weekly sessions of hemodialysis for ≥6 months, using bicarbonate-based dialysate and polysulfone membrane dialyzers in Sichuan Provincial People's hospital, from December 2014 to December 2015. We performed a systematic screening of HBV serological markers by ELISA. The markers included HBsAg(Human HBsAg ELISA Kit, with the sensitivity of 0.1ng/ml, PerkinElmer Inc. China), HBsAb(Human HBsAb ELISA Kit, with the sensitivity of 10IU/L, PerkinElmer Inc. China) and HBcAb(Human HBsAg ELISA Kit, with the sensitivity of 1IU/L, PerkinElmer Inc. China). Collected serum samples from MHD patients with HBsAg(-) /HBcAb(+) and tested for HBV-DNA by real-time PCR assay. After 2 years follow-up OBI patients, samples with persistent HBV-DNA were tested with the S regions by nested PCR, plus sequencing analysis to investigate the genotype of OBI in MHD patients and the gene mutations of “a” determinant of HBsAg. All participants have signed informed consent, and the human work was approved by the Hospital Human Research Ethics Committee of Sichuan Academy of Medical Sciences & Sichuan Provincial People's Hospital.

### Definition of HBV immune status

OBI is defined as the presence of HBV-DNA in the liver or serum with undetectable HBsAg with or without HBV antibodies, and serum HBV-DNA level should be less than 200 IU/ml 13. OBI is most frequently seen in patients with HBsAg(-)/HBcAb(+)/HBsAb(-) (isolated HBcAb+). The presence of HBcAb, but absence of HBsAg, is considered as past HBV infection, including HBsAg(-)/HBcAb(+)/HBsAb(+) and HBsAg(-)/HBcAb(+)/HBsAb(-). Chronic HBV infection is referred to patients who were HBsAg(+)/HBcAb(+) for over at least 6 months. Patients with HBsAb positive and the HBsAb levels>10IU/L were defined as HBV immune via vaccination. And patients with all the HBsAg, HBsAb and HBcAb negative were considered as non-immune to HBV.

### Amplification of HBV S-ORF and Sequencing Assay

HBV-DNA examination: HBV-DNA was extracted using Pure link Viral DNA mini kit (Thermo fisher, USA) according to the manufacturer's instructions. HBV viral load (IU/mL) was measured using real-time polymerase chain reaction (real-time PCR) by COBAS AmpliPrep and COBAS TaqMan HBV Test (Roche, USA) according to the standard manufacturer's instructions, the detection limit of the kit is 20IU/mL according to the user manual 14.

The most conserved regions of S gene sequences were amplified by nest PCR [Prime STAR Max DNA Polymerase (R045A, Takara, Japan)] according to the protocol described. The design of the primer is referred to sequence AF2865942, and the target is the surface open reading frame (S ORF) (Table.1) 15. PCR products were analyzed by electrophoresis on a 1.2% agarose gel.

Amplified products were sequenced by Tsingke Biological Technology from China, and amino acid sequences were aligned with standard sequences from PubMed.

### Statistical analysis

All data were analyzed using SPSS 19. 0 software (IBM Corp, NY, USA). Measurement data is expressed as mean ± SD. Categorical variables were demonstrated as absolute number and percentage.

## Results

### Prevalence of OBI in MHD patients

**Fig 1:**
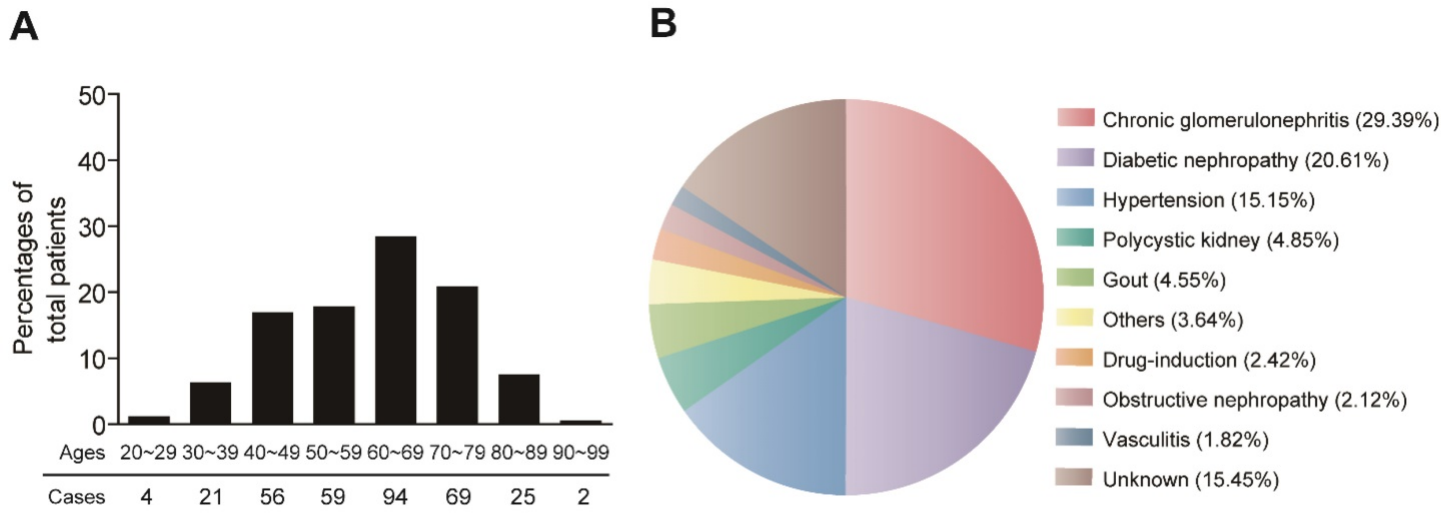
Demographic characteristics of MHD patients.

A total of 330 MHD patients were involved this study. The demographic characteristics of the MHD patients are shown in **Figure 1**. The patients were aged from 27 to 95 years old (60.66±14.32), with majority of patients > 60 years old. The age distribution was similar for female (n=145, 44.3%) and male (n=185, 55.7%) patients (**Figure 1A**). The major causes of MHD patients were chronic glomerulonephritis (29.39%), diabetic nephropathy (20.61%) and hypertensive nephrosclerosis (15.15%) (**Figure 1B**).

All the MHD patients were examined for serological markers of HBV infection. As shown in **Table 2**, we tested the HBsAg, HBcAb and HBsAb in these patients. Results showed that 24(7.3%) of the 330 patients were HBsAg(+) and HBcAb(+) for over at least 6 months indicating chronic HBV infection. A total of 180 patients (54.5%,180/330) were HBsAg(-) but HBcAb(+) and considered to have past HBV infection. Among these 180 patients, 147 patients showed HBsAg(-)/HBcAb(+)/HBsAb(+), suggesting that they were exposed to HBV in the past and developed natural immunity to HBV. The other 33 patients showed HBsAg(-)/HBcAb(+)/HBsAb(-)(isolated HBcAb+), which were exposed to HBV in the past but did not develop natural immunity to HBV. 15.5% (51/330) patients were only HBsAb(+) and the levels of >10IU/L, probably due to previous vaccination. Moreover, there were 21.8% (72/330) patients showed HBsAg(-)/HBcAb(-)/HBsAb(-) ,indicating non-immune to HBV, and 0.9% (3/330) patients were HBsAg(+)/HBcAb(+)/HBsAb(+), suggesting that they have early (acute) HBV infection (**Table 2**).

Next, we investigated the prevalence of OBI in these MHD patients. So we focused on the 180 patients with HBsAg(-)/HBcAb(+)( past HBV infection). We ultimately collected 165 plasma samples for further evaluation of OBI by examining the HBV-DNA using real-time PCR. Results revealed that there were 7 patients showed HBV-DNA positive, indicating that they were OBI. It's worth noting that all of these 7 OBI patients were male. Moreover, five patients (No. 1, 3, 4, 5 and 7) were HBsAg(-)/HBcAb(+)/HBsAb(+), and 2 patients (No. 2 and 6) were isolated HBcAb (**Table 3**). Therefore the prevalence of OBI was 4.2% (7/165) in the test group, and 2.1% (7/330) in the overall dialysis cohort.

Then, we followed up these 7 patients for 2 years, to re-examine their HBV-DNA. Unfortunately, No. 3 and 4 patients were deceased. Interestingly, samples from 3 patients that were HBsAg(-)/HBcAb(+)/HBsAb(+), were not able to detect HBV-DNA. Only patients No. 2 and 6, which were isolated HBcAb(+), still have detectable HBV-DNA after 2 years. This result indicated the OBI patients with isolated HBcAb(+) would have sustained HBV-DNA (**Table. 3**).

### Molecular characterization and analysis in the “a” determinant of HBsAg

We next explored the underlying mechanism of the OBI in our study. Previous studies established that mutations in the “a” determinant of HBsAg was one of the earliest recognized mechanisms leading to OBI, which lead to conformational changes rendering the protein undetectable by commercial assays 10. Therefore, we analyzed the genomic mutations in No. 2 and 6 patient mentioned above with OBI. By nested PCR and sequencing analysis, we found that these two patients were genotype B.

**Figure 2:**
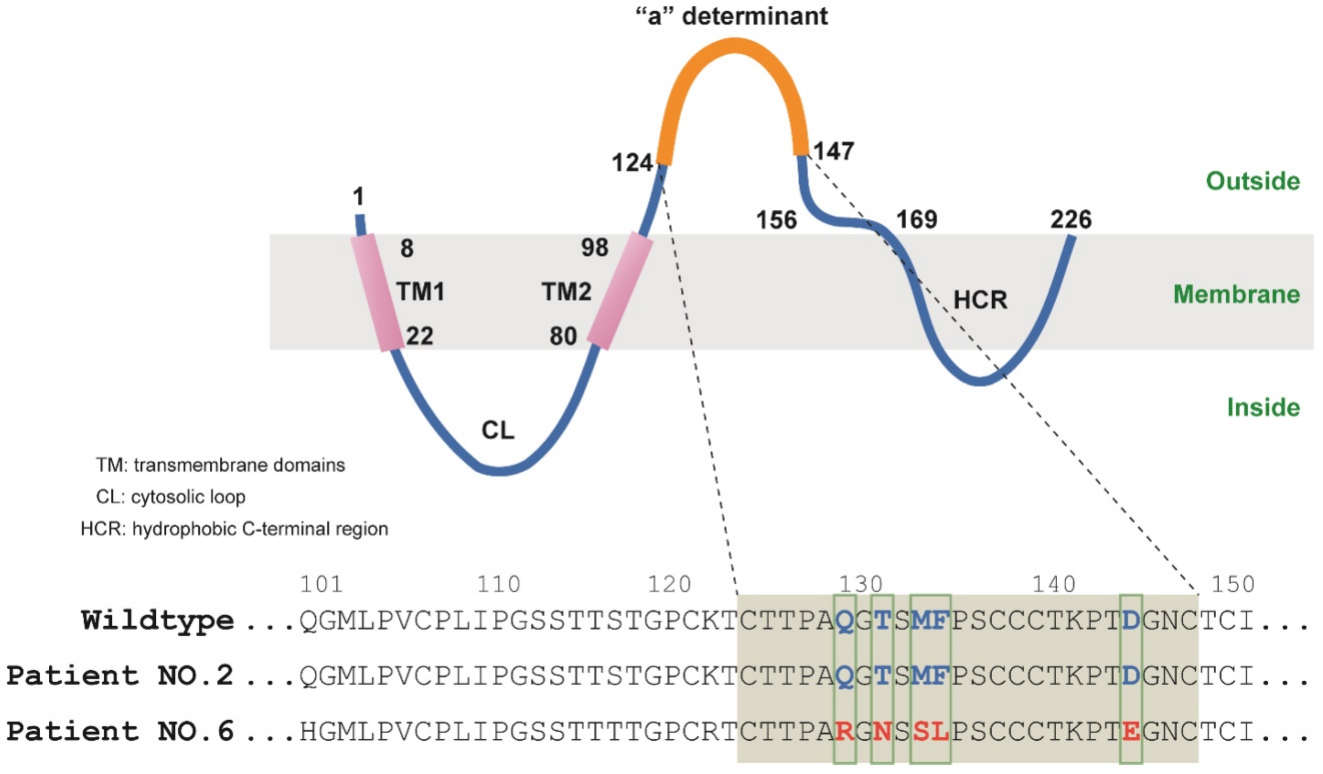
Mutations in the “a” determinant of HBV S gene.

And further amino acid analysis revealed that patient No.2 did not carry any amino acid mutation in the “a” determinant. However, patient No.6 was found to have mutations of Gln129Arg (Q129R), Thr131Asn (T131N), Met133Ser (M133S), Phe134Leu (F134L) and Asp144Glu (D144E) in the “a” determinant of HBsAg (**Figure 2, Table 3**). Therefore, these mutations may be responsible for the escape of HBsAg detection.

## Discussion

Hepatitis B virus (HBV) infection is one of the major infectious diseases with >250 million chronic carriers worldwide 16. Currently, HBV transmission has been controlled in hemodialysis centers by universal precautions and vaccination, however, MHD patients still are at risk of acquiring HBV, especially for OBI 17. Manoochehr et al. recommended that MHD patients should take OBI screening 18. According to the references, the prevalence of OBI among MHD patients ranges from 0% to 58% in the world 9. In our study, we assessed the prevalence of OBI in MHD patients in Sichuan Province, Southwest of China firstly. We found that the prevalence of chronic HBV infection was 7.3% in the center, the prevalence of OBI was 4.2% (7/165) in the test group, and 2.1% (7/330) in the overall dialysis cohort. The prevalence is likely underestimated, as 15 patients didn't take the HBV-DNA test, and it is possible there are still OBI patients in those who didn't have HBV infection in the past. Nevertheless, the prevalence is higher than that in the MHD patients of UK as Sowole reported (0.4%) 13. Sowole et. al. showed that the past HBV rate was 20% and isolated HBcAb(+) was 3%. In our cohort, the past HBV rate was 54.5% and the isolated HBcAb(+) was 10%. As OBI is most frequently seen in patients with isolated HBcAb(+), we assumed that the OBI rate in our cohort may be higher than that in UK.

OBI is a clinical class of HBV infection and can appear in two forms: seropositive OBI and seronegative OBI. seropositive OBI: HBcAb has a positive outcome with or without HBsAb positive. seronegative OBI: both HBsAb, HBcAb were negative. In general, about 20% of individuals with OBI are seronegative OBI, and 80% of individuals with OBI are seropositive OBI 19. In this study, all the 7 OBI patients with MHD are seronegative, which is in accordance with the theory that OBI is more prevalent in seronegative patients 20. The 7 OBI patients with MHD were followed up for 2 years, 2 of them were dead in the period. 3 of the HBsAg(-)/HBcAb(+)/HBsAb(+) patients were found HBV-DNA negative, meanwhile the HBV-DNA can still be detected in the 2 of the isolated HBcAb(+) patients. The possible explanation is HBcAb constitutes the first antibody responsed to HBV infection, which is non-protective antibody that couldn't neutralize or suppress HBV virus. In contrast, HBsAb is the only HBV serologic marker that can be detected after vaccination against this virus, and appears late in infection after the disappearance of HBsAg and has a protective neutralizing effect, which neutralize the HBV virosome, and disable its ability to infect 20. Furthermore, we noticed the HBV-DNA level in the 2 isolated HBcAb(+) patients is detectable, but lower than 20IU/ml. Interestingly, as is mentioned in previous references, in more than 90% of OBI patients, the viral load in serum was reported to be around 20 IU/mL 18. Although the HBV-DNA level in OBI patients with MHD is low, the presence can be detected for a long time. It's reported that the persistence of OBI may lead the patients to the risk of progression to liver disease, development of hepatocellular carcinoma, cirrhosis and occurrence of fulminant hepatitis, these patients are able to transmit infection to others through dialysis, blood transfusion, etc 9. Different genotypes are associated with different clinical outcomes. HBV can be divided into genotypes from A to J based on the divergence of the complete genome sequences by 8% or more, or the divergence of the S gene sequences by 4% or more. The HBV genotype A and D are common in Western countries, while the HBV genotype B and C are common in Asia areas including China 21. It's reported that genotype B is more likely to disseminate in hemodialysis environmental 22. In this study, the results showed all of the OBI patients are genotype B, which is accordant with the previous reports 22.

While the exact mechanism of OBI is unclear, several explanations have been proposed. One probable theory is genetic variations in the“a” determinant of HBsAg, which inhibit HBsAg production and viral replication 18. Different studies showed numerous mutations in the HBV genome of OBI patients. According to the reports, the “a” determinant of OBI patients contains Gly145Arg/Ala/Ile/Trp/Glu (G145R/A/I/W/E), Leu109Arg (L109R), Gly119Arg (G119R), Pro120Thr (P120T), Lys122Ile (K122I), Thr126Asn (T126N), Thr127Arg (T127R), Gln129Asn (Q129N), Thr131Asn (T131N), Met133Thr/Leu (M133T/L), Phe134Leu (F134L), Cys139Ser (C139S), Thr140Leu (T140L), Asp144Glu (D144E) and Pro153Leu (P153L) mutants 23-25. Which leads to the production of different surface antigen and escape mutants. We found Gln129Arg (Q129R), Thr131Asn (T131N), Met133Ser (M133S), Phe134Leu (F134L), Asp144Glu (D144E) mutants in the “a” determinant of the S gene in one of the patients. The presence of Thr131Asn (T131N), Phe134Leu (F134L) and Asp144Glu (D144E) mutants was showed in other studies. Therefore, it's the first time Gln129Arg (Q129R) and Met133Ser (M133S) mutants are reported.

However, there still exist some limitations in this study. Firstly, we focused on the 180 patients with HBsAg(-)/HBcAb(+)( past HBV infection), and 15 patients of them didn't take the HBV-DNA test, and it is possible there are still OBI patients in those who didn't have HBV infection in the past. Therefore, the rate of OBI may be under-estimated. Moreover, the mechanistic study of how Q129 and M133 mutations causes OBI was not detected in current study. We would like to assess the functional implication of these two mutations in future work.

## Conclusion

In this study, we assessed the prevalence of OBI in MHD patients in Sichuan Province of China firstly. The prevalence of OBI was 4.2% (7/165) in the test group, and 2.1% (7/330) in the overall dialysis cohort. In the molecular analysis, we for the first time demonstrate the mutations of Gln129Arg (Q129R) and Met133Ser (M133S) in the “a” determinant of HBsAg in an OBI patient. This work clarifies the prevalence of OBI in MHD patients in Sichuan Province, and provides the molecular characterization of the occult HBV infection.

## Declarations

### Availability of data and materials

The datasets used and/or analyzed during the current study are available from the corresponding author on reasonable request.

### Funding

This work was supported by Chinese Hospital Association Foundation (No. CHABP2016-12) and Wu Jieping Medical Foundation (No. 320.6750.16193).

### Author Contributions

Y.T. analysed the experimental data, and was a major contributor in writing the manuscript. X.Q.L. examined the serological assessment. X.H.L. collected information of patients and analyzed patients information. Q.H. and G.L. collected samples from patients. Y.Z. designed and fund this work. All authors read and approved the final manuscript.

## Figures and Tables

**Table 1:**
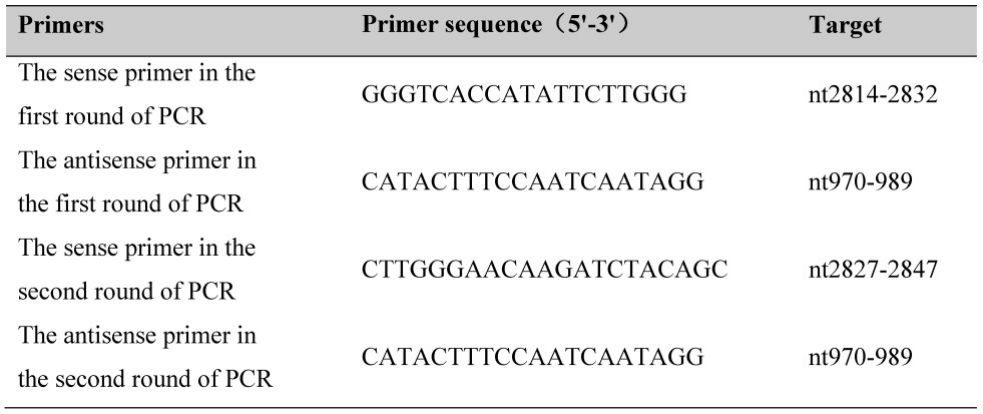
Amplification Assay of HBV S-ORF.

**Table 2:**
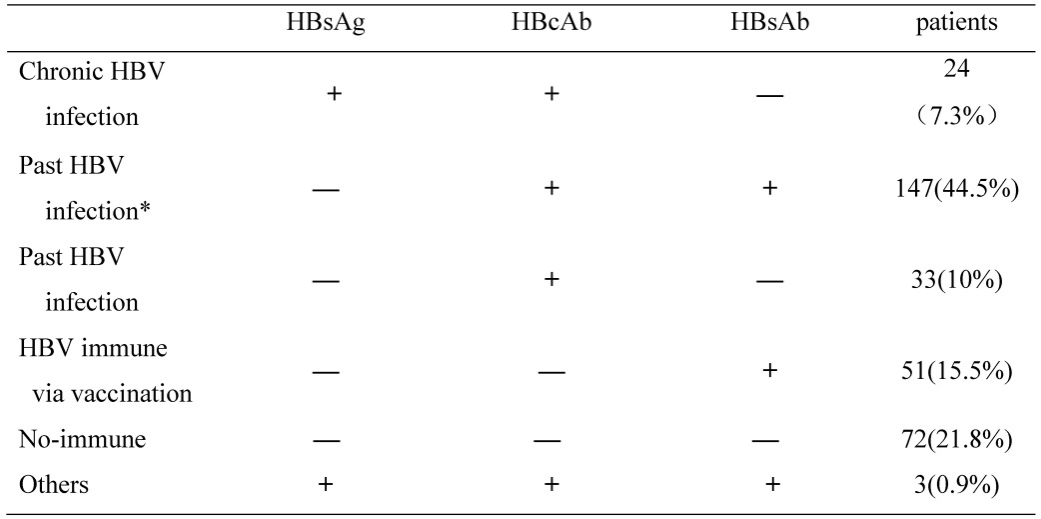
Characterization of HBV infection in MHD patients.

HBV, Hepatitis B virus; HBsAg, Hepatitis B surface antigen; HBcAb, hepatitis B Core antibody; HBsAb, hepatitis B surface antibody. * Patients who have acquired natural immunity due to prior exposure to hepatitis B.

**Table 3:**
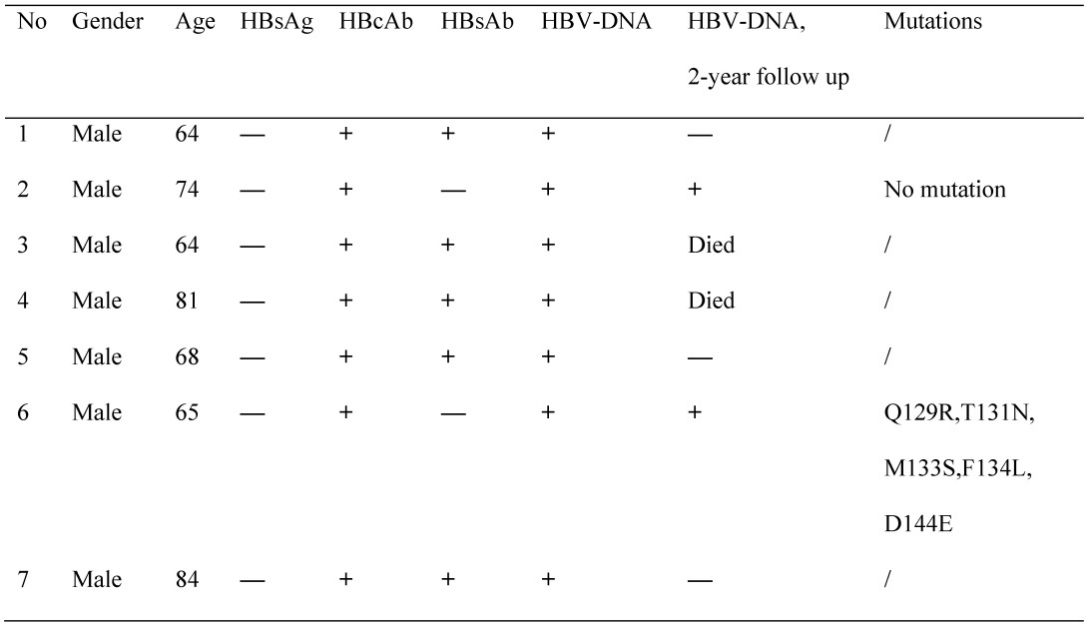
Characterization of OBI patients

OBI, occult HBV infection; HBV, Hepatitis B virus; HBsAg, Hepatitis B surface antigen; HBcAb, hepatitis B Core antibody; HBsAb, hepatitis B surface antibody;+:positive;—:negative;/:untested; Q129R :Gln129Arg, T131N :Thr131Asn, M133S :Met133Ser, F134L :Phe134Leu, D144E :Asp144Glu
